# Multiple metals exposure and estimated pulse wave velocity: a cross-sectional analysis of the US adults

**DOI:** 10.3389/fpubh.2025.1606518

**Published:** 2025-08-26

**Authors:** Shijie Zhou, Zhihao Xiao, Boyu Shi, Zuqiang Fu, Shourui Wang, Wenxiang Li, Zhiwei Liu, Qian Liu, Xinxin Gu, Aihua Gu

**Affiliations:** ^1^State Key Laboratory of Reproductive Medicine and Offspring Health, School of Public Health, Nanjing Medical University, Nanjing, China; ^2^Jiangsu Environmental Health Risk Assessment Engineering Research Center, Key Laboratory of Modern Toxicology of Ministry of Education, Center for Global Health, Nanjing Medical University, Nanjing, China; ^3^School of Public Health, Southeast University, Nanjing, China; ^4^Tianyuan Honors School, Nanjing Medical University, Nanjing, China; ^5^Qidong People’s Hospital, Qidong, China

**Keywords:** multiple metals exposure, ePWV, NHANES, WQS, Qgcomp, mediation analysis

## Abstract

**Background and objectives:**

Arterial stiffness has been demonstrated to be associated with a range of adverse cardiovascular events. Nevertheless, the epidemiological evidence on the association between metal exposure and arterial stiffness remains inconclusive.

**Methods:**

The data concerning 12 urine metals were derived from the National Health and Nutrition Examination Survey (NHANES) conducted from 2003 to 2016. Multiple linear regression and restricted cubic spline (RCS) analyses were applied to explore the potential linear and nonlinear associations between urine metal and ePWV. A parallel mediation analysis was conducted in order to explore the potential intermediate factors in metal-induced ePWV elevation. Weighted quantile sum (WQS) regression and Quantile g-computation (Qgcomp) were conducted to estimate the individual and combined associations between urine metal and ePWV.

**Results:**

Following adjustment for the relevant covariates, it was found that urine Cd, Pb, Co, and U were found to be significantly correlated to elevated ePWV in both the multiple linear regression and the RCS model. Mediation analysis revealed that high - density lipoprotein (HDL) and total cholesterol (TC) might be partly implicated in the correlation between urine metal and ePWV. WQS regression and Qgcomp analyses consistently indicate a positive correlation between exposure to mixed metals and elevated ePWV, with Cd and Pb identified as the primary contributors to this phenomenon.

**Conclusion:**

The present study indicated a significant association between the presence of a mixture of metals and elevated ePWV, with Cd and Pb identified as the primary risk factors. And HDL and TC might participate in mediating mixed metals exposure induced ePWV changes.

## 1 Introduction

Arterial stiffness is a phenotype that appears early in the development of many cardiovascular diseases. Therefore, arterial stiffness is accounted as a predict index of several adverse cardiovascular outcomes such as hypertension, atherosclerosis and stroke (1–3). Pulse wave velocity (PWV) has been recommended as a non-invasive means of assessing arterial stiffness. This, in turn, can be used to evaluate organ damage resulting from arterial hypertension (4–7). In 2016, Greve et al. derived estimate pulse wave velocity (ePWV) based on carotid-femoral pulse wave velocity (cfPWV) and demonstrated that ePWV has better predictive value in healthy patients and untreated hypertensive patients (8). And ePWV was gradually recognized for its reliability and low threshold. Vlachopoulos et al. have utilized ePWV as a metric to evaluate the incidence of adverse cardiovascular outcomes, including stroke, coronary heart disease and cardiovascular death, in individuals at risk of cardiovascular disease (CVD) within the SPRINT subgroups (9). Solini et al. also established that in patients diagnosed with type 2 diabetes, elevated level of ePWV indicated a lower survival rates of patients with cardiac and renal complications (10).

Metals are widely used in storage batteries, electroplating industry, chemical raw materials and other industries (11). With the progress of industrialization and urbanization, a mass amount of metals are discharged into the environment in the approach of mining, smelting, exhaust emissions and sewage irrigation, which are contacted and introduced into the human body through drinking water and crops, and then accumulated in the human body (12–14). Metals are generally considered to affect a range of systems and organs, including the nervous system (15–17) and hematopoietic system (18–20). Recent years have seen an increased focus on the association between metal exposure and cardiovascular disease risk. However, there is a paucity of research on the association between metal exposure and arterial stiffness, and the conclusions vary (21, 22). Therefore, the potential role of metal exposure in arterial stiffness remains understudied.

In this study, we extracted data pertaining to urine metal and other sociodemographic characteristics of the population during 2003–2016 cycle, and then calculated ePWV based on age and blood pressure. The relationship between urine metals and ePWV were investigated through several different statistical strategies. In addition, we also investigated which potential factors could mediates the association between urine metal levels and ePWV.

## 2 Materials and methods

### 2.1 Study population

The data utilized in this study has been drawn from the NHANES database. The latter is a cross-sectional study conducted on a nationwide scale, overseen by the National Center for Health Statistics. The survey enables researchers to obtain information related to demographics, socioeconomics, diet, health, and physical examination from a sample of the US population that is representative of the whole population. The survey data can then be utilized to appraise the health and nutritional status of the population. In summary, from 2003 to 2016, relevant indicators were collected from approximately 70,000 U.S. residents. All data and indicator sources for the study can be referred to on public access.^1^

**Figure 1:**
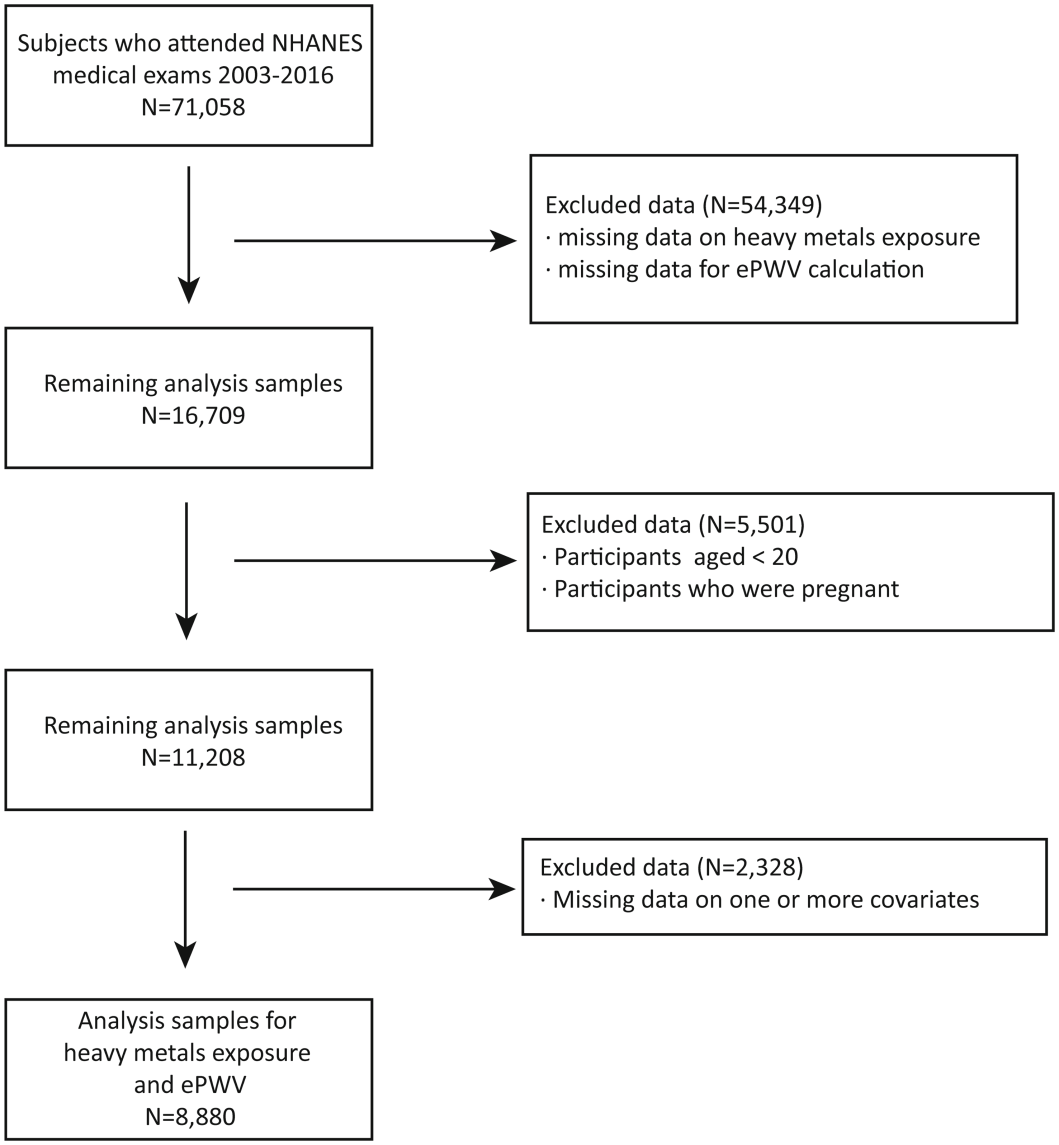
Flow diagram for participant inclusion.

Among the 71,058 subjects available for the study, the following conditions were set for data cleaning: (1) lack of data on metal exposure and data required for calculation of the ePWV index; (2) subjects under the age of 20 or pregnant; (3) missing one or more covariates. Eventually, 8,800 samples were included in the analysis to verify the association between metal exposure and ePWV. The detailed flow chart of the included subjects is shown in Figure 1.

### 2.2 Acquisition of estimated pulse wave velocity (ePWV)

The formula for ePWV is referenced in Greve et al. and is derived from the collaborative reference value for arterial stiffness (8). ePWV = 9.587–0.402 × age + 4.560 × 10^−3^ × age^2^–2.621 × 10^−5^ × age^2^ × MBP + 3.176 × 10^−3^ × age × MBP - 1.832 × 10^−2^ × MBP. MBP was calculated by the following formula: (DBP) + 0.4 (SBP − DBP). And ePWV was trisected as T1 (<6.88 m/s), T2 (6.88–9.20 m/s), T3 (>9.20 m/s).

### 2.3 Measurement of metals in urine

Urine metal levels are favored as a noninvasive assay for large follow-up cohorts compared to serum metal levels. According to the guiding manual, the urine samples were pretreated, stored on appropriate medium and transported to the laboratory for analysis. Urine levels of 12 selected metals [including Barium (Ba), Cobalt (Co), Cesium (Cs), Molybdenum (Mo), Antimony (Sb), Tungsten (W), Uranium (U), Thallium (Tl), Lead (Pb), Cadmium (Cd), Mercury (Hg), Arsenic (As)] were measured by inductively coupled plasma mass spectrometry (ICP-MS) analysis (23). Based on these results, we extracted data of urine metal levels in the period 2003 to 2016, and all 12 metals were detected at a rate of more than 60%, and were corrected by creatinine (Supplementary Table S1). The distribution of these metals among the study participants can be seen in Supplementary Table S2.

### 2.4 Covariates

In accordance with the previous literature, the core covariates selected for this study are outlined below: sociodemographic characteristics, including sex (male/female), age (under 40 years old/40–60 years old/over 60 years old), race (Mexican American/other Hispanic/non-Hispanic White/non-Hispanic Black/other), education level (less than 11th grade/ high school grade/ some college or above), poverty income ratio (PIR); life style and body measurement indicators, including physical activity, body mass index (BMI), TC, HDL, white blood cell counts; alcohol consumption, smoking status, total energy intake and urine creatinine. PIR (%) was divided into categorical variable according to the quartile [Q1 (<1.18), Q2 (1.18–2.20), Q3 (2.21–4.10), Q4 (>4.10)]. Physical activity was divided into three categories based on the questionnaire (never/moderate/vigorous). Smoking status was (never/former/current) based on cigarettes consumption (more than 100) and whether smoking currently. Drinking status was based on alcoholic beverage consumption. The conversion of alcohol consumption was in accordance with the previous method. The results were “No” and “Yes.” BMI was coded as continuous variables (24). Considering the correlation between arterial stiffness and related disease, we additionally regarded diabetes, hypertension, and CVD as covariates in our analysis (25).

### 2.5 Statistical analysis

The mean ± standard deviation (SD) was utilized to depict continuous variables conforming to a normal distribution, in order to provide descriptive information on the sociodemographic characteristics. Other variables were defined as categorical variables and are expressed as *n* (%). In this study, metal concentrations (μg/mg) were adjusted by urine creatinine to analyze the correlation between urine metal and ePWV. In order to circumvent the potential bias engendered by a skewed distribution, the concentrations of urine metals were transformed through log10 function in order to achieve approximate normal distribution (continuous variable), or categorized into quartiles (Q1, Q2, Q3, and Q4) as categorical variables. The internal relevance between the mixed urine metals was evaluated by Spearman’s correlation coefficient.

Multiple linear regression model was utilized to investigate the association between each urine metal and ePWV. Mediation analysis was performed to ascertain the mediating effect of serum lipid levels and white blood cell counts on the associations between urine metals and ePWV. Considering the complexity of real environmental exposure, WQS analysis was employed to investigate the joint effects of mixed metal exposures on ePWV. And the contribution of every single metal to the holistic indices effect can then be assessed through the weight assigned to each variable by the model. Additionally, we introduced the Qgcomp model to confirm the effects of each urine metal on ePWV. This was achieved by allocating positive or negative weights to each factor in the model. Restricted cubic spline (RCS) regression was employed to estimate the potential nonlinear relationship between each urine metal and ePWV. Spearman’s correlation coefficient was employed to examine relationships between urine metals.

All statistical methods have been adjusted for the aforementioned confounding variables. These analyses were executed utilizing the R software platform (R 4.1.1). In the statistical model, significance was affirmed by a two-sided *p*-value of under 0.05.

## 3 Results

### 3.1 Baseline characterization

The basic characteristics of the participants which were involved in study are shown in Table 1. A total of 71,058 participants were collected for this study and the final number included in the analysis was 8,880, including 4,585 male and 4,295 female. Higher level of ePWV was prefer to exist in the subgroup which participants over 60 years old, male, of Non-Hispanic White, have lower level of education, with less exercise, had a history of smoking. Additionally, we observed the higher levels of ePWV were accompanied by a higher prevalence of hypertension, CVD, diabetes.

**Table 1:** Baseline characteristics of the participants in the analyses.

Characteristics	Total population	ePWV (m/s)		
		T1 (<6.88)	T2 (6.88–9.20)	T3 (>9.20)
Overall	8,880	2,931	2,930	3,019
Age (years, %)				
<40	2,857 (32.2)	2,402 (82.0)	453 (15.5)	2 (0.1)
40–60	2,957 (33.3)	523 (17.8)	2,102 (71.7)	332 (11.0)
>60	3,066 (34.5)	6 (0.2)	375 (12.8)	2,685 (88.9)
Gender (%)				
Male	4,585 (51.6)	1,415 (48.3)	1,565 (53.4)	1,605 (53.2)
Female	4,295 (48.4)	1,516 (51.7)	1,365 (46.6)	1,414 (46.8)
Race (%)				
Mexican American	1,413 (15.9)	565 (19.3)	479 (16.3)	369 (12.2)
Other Hispanic	754 (8.5)	278 (9.5)	250 (8.5)	226 (7.5)
Non-Hispanic White	4,238 (47.7)	1,246 (42.5)	1,361 (46.5)	1,631 (54.0)
Non-Hispanic Black	1785 (20.1)	553 (18.9)	617 (21.1)	615 (20.4)
Other	690 (7.8)	289 (9.9)	223 (7.6)	178 (5.9)
Education level (%)				
Less Than 11th Grade	2,189 (24.7)	592 (20.2)	643 (21.9)	954 (31.6)
High School Grad	2064 (23.2)	655 (22.3)	685 (23.4)	724 (24.0)
Some College or above	4,627 (52.1)	1,684 (57.5)	1,602 (54.7)	1,341 (44.4)
Physical activity (%)				
Inactive	4,760 (53.6)	1,416 (48.3)	1,493 (51.0)	1851 (61.3)
Moderate	2,158 (24.3)	677 (23.1)	706 (24.1)	775 (25.7)
Vigorous	1962 (22.1)	838 (28.6)	731 (24.9)	393 (13.0)
Past-year alcohol drinking (%)				
No	2,466 (27.8)	693 (23.6)	760 (25.9)	1,013 (33.6)
Yes	6,414 (72.2)	2,238 (76.4)	2,170 (74.1)	2006 (66.4)
Smoking status (%)				
Never	4,715 (53.1)	1758 (60.0)	1,545 (52.7)	1,412 (46.8)
Former	2,353 (26.5)	405 (13.8)	709 (24.2)	1,239 (41.0)
Current	1812 (20.4)	768 (26.2)	676 (23.1)	368 (12.2)
Family PIR (%)				
Q1 (<1.18)	2,112 (23.8)	822 (28.0)	630 (21.5)	660 (21.9)
Q2 (1.18–2.20)	2,211 (24.9)	705 (24.1)	624 (21.3)	882 (29.2)
Q3 (2.21–4.10)	2,261 (25.5)	727 (24.8)	742 (25.3)	792 (26.2)
Q4 (>4.10)	2,296 (25.9)	677 (23.1)	934 (31.9)	685 (22.7)
Total energy intake (kcal, %)				
Q1 (<1472.0)	2,124 (23.9)	530 (18.1)	623 (21.3)	971 (32.2)
Q2 (1472.0–1915.0)	2,230 (25.1)	670 (22.9)	676 (23.1)	884 (29.3)
Q3 (1915.1–2502.5)	2,238 (25.2)	757 (25.8)	751 (25.6)	730 (24.2)
Q4 (>2502.5)	2,288 (25.8)	974 (33.2)	880 (30.0)	434 (14.4)
CVD (%)				
No	7,913 (89.1)	2,874 (98.1)	2,717 (92.7)	2,322 (76.9)
Yes	967 (10.9)	57 (1.9)	213 (7.3)	697 (23.1)
Hypertension (%)				
No	5,673 (63.9)	2,661 (90.8)	1875 (64.0)	1,137 (37.7)
Yes	3,207 (36.1)	270 (9.2)	1,055 (36.0)	1882 (62.3)
Diabetes (%)				
No	7,608 (85.7)	2,830 (96.6)	2,470 (84.3)	2,308 (76.4)
Yes	1,272 (14.3)	101 (3.4)	460 (15.7)	711 (23.6)
BMI (kg/m^2^)	29.04 ± 6.67	27.78 ± 6.68	30.32 ± 6.88	29.01 ± 6.20
HDL (mg/dL)	53.05 ± 16.16	52.54 ± 14.78	51.70 ± 16.44	54.86 ± 16.99
TC (mg/dL)	195.62 ± 42.09	185.94 ± 37.97	202.58 ± 42.85	198.26 ± 43.43

All data originated from NHANES 2003–2016. Data are presented as frequencies (%) for categorial variables, and mean ± geometric standard deviation continuous variables.

ePWV, Estimated Pulse Wave Velocity. T, Tri-sectional quantile. Q, quartile. Family PIR, ratio of family income to poverty. CVD, Cardiovascular diseases. BMI, Body mass index. HDL, High-Density Lipoprotein. TC, Total Cholesterol.

### 3.2 Spearman’s correlation coefficient for the mutual correlations among the urine metals

In order to investigate mutual correlations among the environmental metal exposures, we calculated Spearman’s correlation coefficients to check the correlation between pairwise urine metals. Positive but weak correlations were observed between almost all of the 12 metals in urine (Supplementary Figure S1). The highest levels of correlation were observed between U and Ba (*r* = 0.21, *p* < 0.05), Tl and Cs (*r* = 0.2, *p* < 0.05).

### 3.3 Associations between urine metals and ePWV

**Figure 2:**
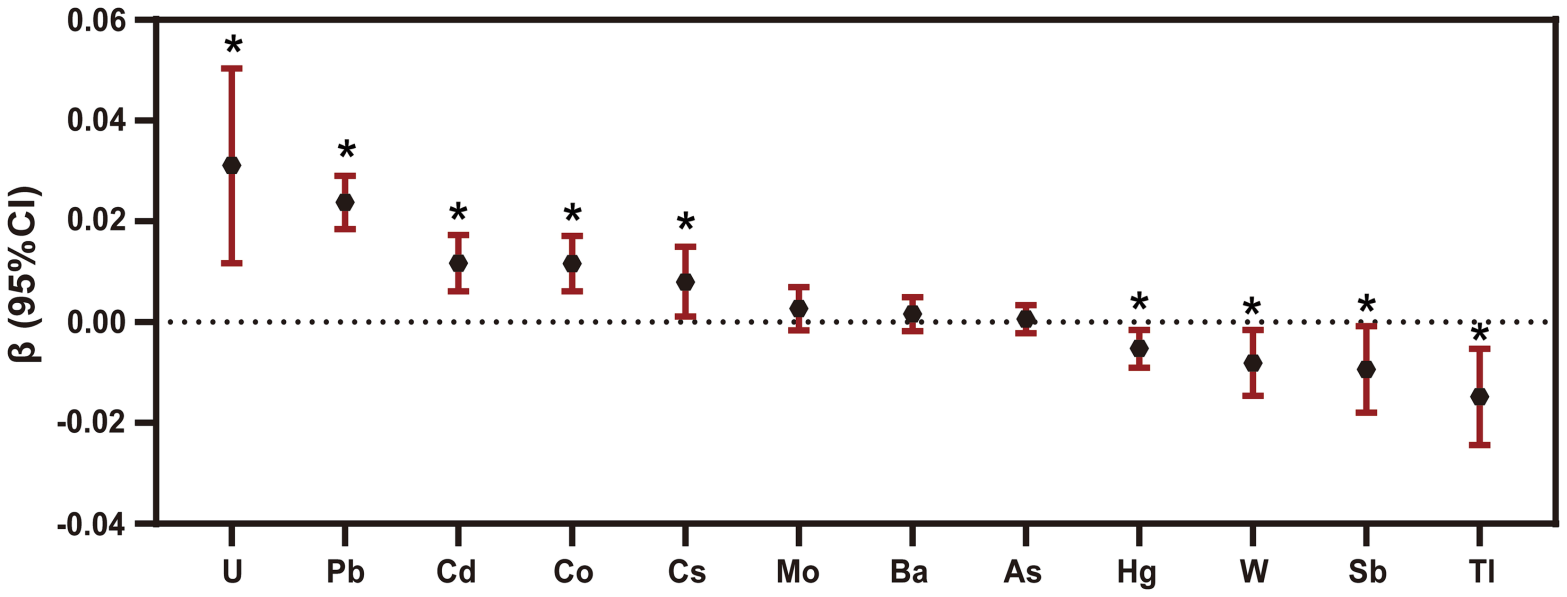
The association between environmental metal exposures with ePWV. The multiple linear regression models were adjusted for age, gender, race, education levels, smoking status, past-year alcohol drinking, intake of total energy, physical activity, BMI, ratio of family income to poverty, diabetes, hypertension, CVD (cardiovascular diseases), NHANES survey circle, level of high-density lipoprotein and total cholesterol. * *p* < 0.05.

According to the results exhibited in Figure 2, Supplementary Table S3, we found that Cd (*β* = 0.012, 95% CI: 0.006, 0.017), Co (*β* = 0.012, 95% CI: 0.006, 0.017), Cs (*β* = 0.008, 95% CI: 0.001, 0.015), Pb (*β* = 0.024, 95% CI: 0.019, 0.029), U (*β* = 0.031, 95% CI: 0.012, 0.051) levels were positively correlated with the ePWV. And an inverse correlation has been observed between Sb (*β* = −0.009, 95% CI: −0.018, −0.001), Tl (*β* = −0.015, 95% CI: −0.024, −0.005), W (*β* = −0.008, 95% CI: −0.015, −0.002), Hg (*β* = −0.005, 95% CI: −0.009, −0.002) and the levels of ePWV (*p* < 0.05).

### 3.4 RCS analysis between multiple metals exposure and ePWV

**Figure 3:**
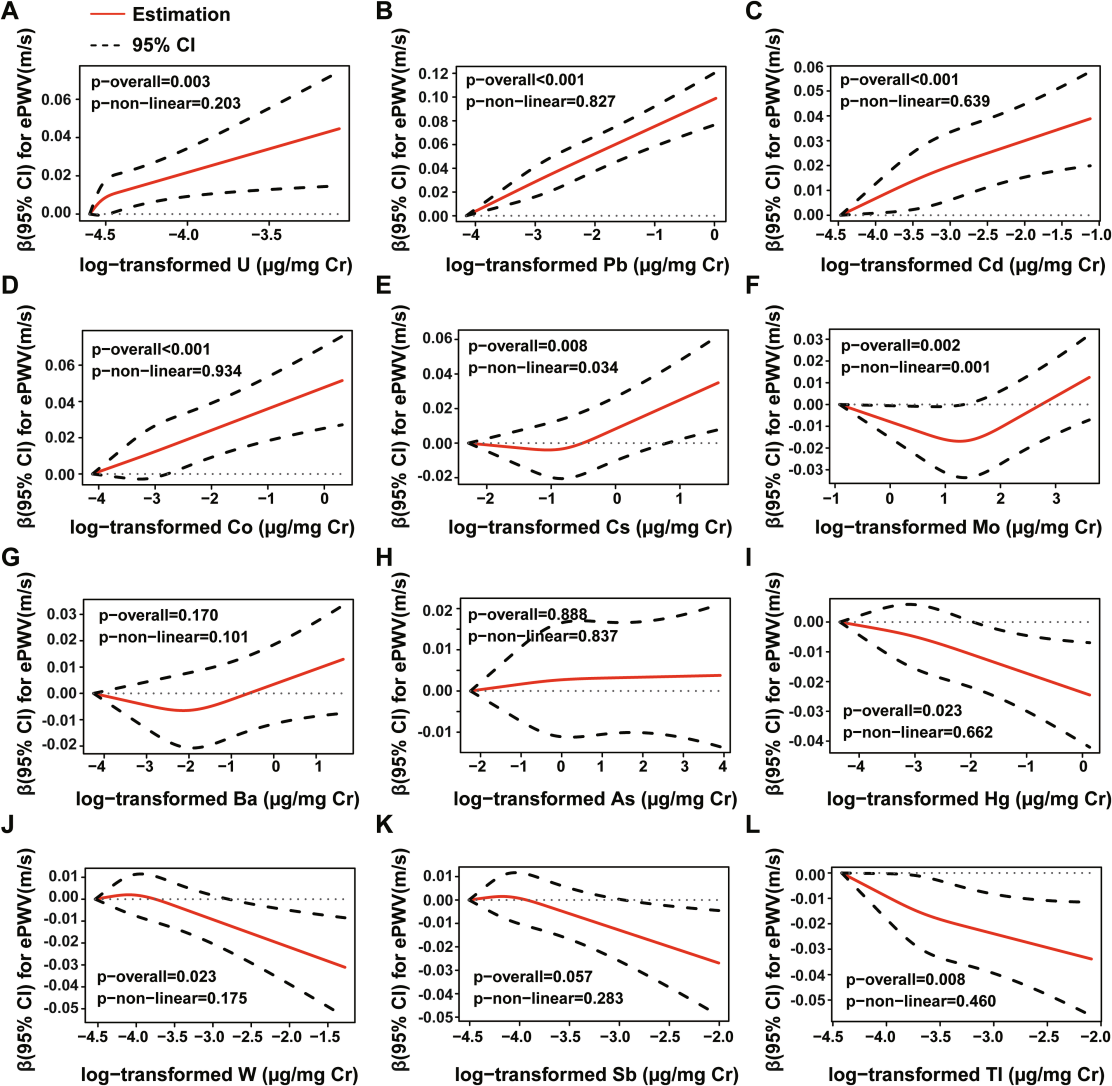
RCS regression between individual urine metal and ePWV. **(A)** U, **(B)** Pb, **(C)** Cd, **(D)** Co, **(E)** Cs, **(F)** Mo, **(G)** Ba, **(H)** As, **(I)** Hg, **(J)** W, **(K)** Sb, **(L)** Tl. The model was adjusted for gender, age, race, education levels, smoking status, past-year alcohol drinking, intake of total energy, physical activity, BMI, ratio of family income to poverty, diabetes, hypertension, CVD, NHANES survey circle, level of high-density lipoprotein and total cholesterol.

We conducted RCS analysis to visualize the potential nonlinear relationships between urine metals and ePWV. As exhibited in Figure 3, nine of all urine metals were significantly associated with ePWV (*p*-overall < 0.05), while U, Pb, Co, Cd showed positive associations with ePWV. The analysis results also revealed that Cs, Mo were positively correlated with the ePWV and in a nonlinear mode (*p*-non-linear < 0.05).

### 3.5 WQS analysis of the effects of multiple and individual metal exposure on ePWV

**Figure 4:**
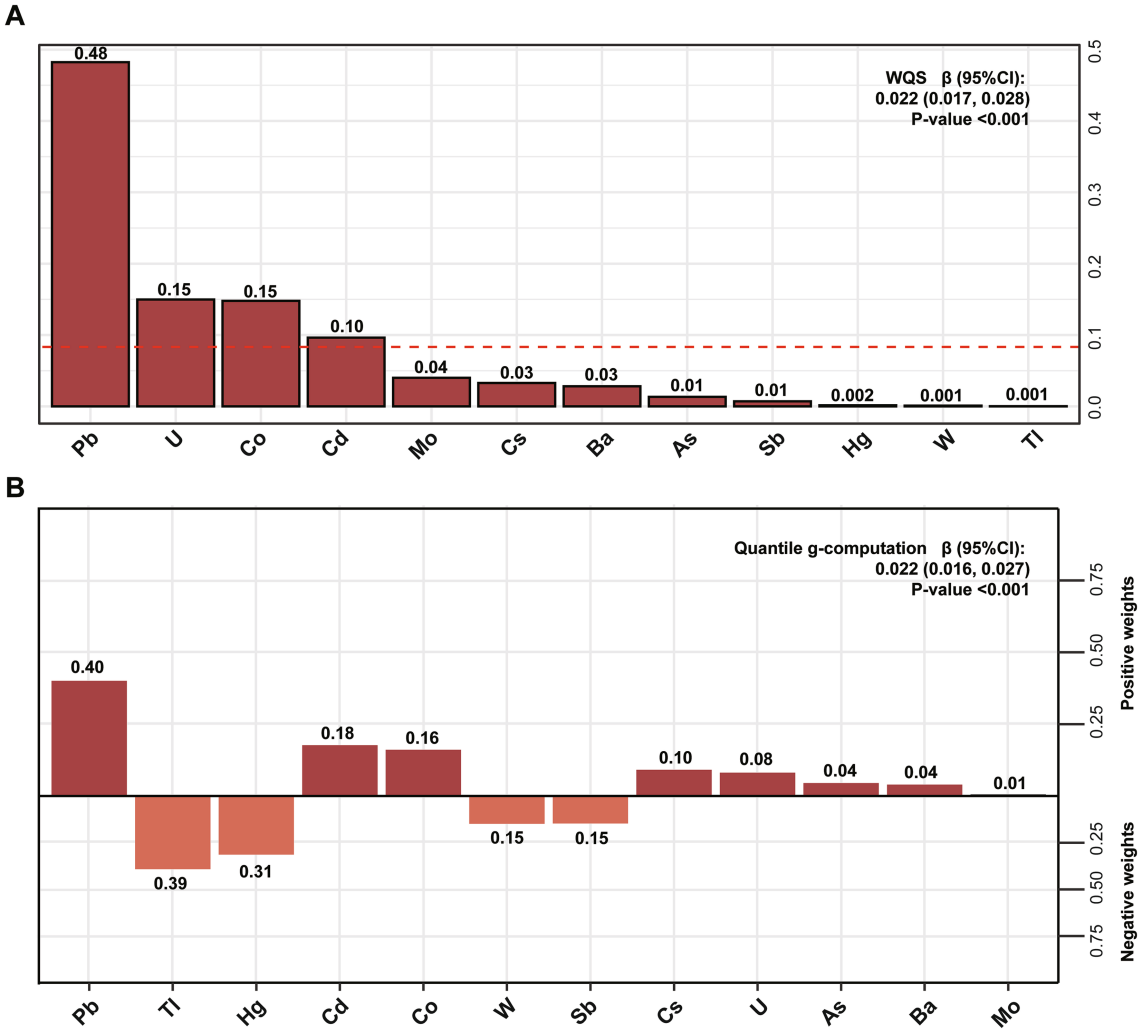
Both the joint and individual effects of urine metals on ePWV were evaluated by WQS **(A)** and Qgcomp **(B)** model. Models were adjusted for gender, age, race, education levels, smoking status, past-year alcohol drinking, intake of total energy, physical activity, BMI, ratio of family income to poverty, diabetes, hypertension, CVD, NHANES survey circle, level of high-density lipoprotein and total cholesterol.

Our findings indicated that the WQS indices exhibited a substantial correlation with ePWV. As shown in Figure 4A, the upper quartile of the WQS indices was significantly related to elevated ePWV (*β* = 0.022, 95% CI: 0.017, 0.028), in which U (*β* = 0.15), Pb (*β* = 0.48), Co (*β* = 0.15), and Cd (*β* = 0.1) were the dominating contributors to the positive correlation (*p* < 0.01).

### 3.6 Qgcomp analysis of the effects of multiple and individual metal exposure on ePWV

Unlike other mixed exposure models, Qgcomp analysis does not require all weight indices to be aligned in the same direction. Our findings showed mixed metals exposure were significantly and positively associated with ePWV (*β* = 0.022, 95% CI: 0.016, 0.027). For the levels of ePWV, the urine level of Pb (*β* = 0.4) showed the strongest positive correlation, followed by Cd (*β* = 0.18), Co (*β* = 0.16) and Cs (*β* = 0.10) (Figure 4B). When it comes to negative relationship between urine metals and ePWV, TI, Hg, W, Sb exhibited a negative tendency with ePWV.

### 3.7 Mediation analysis of potential intermediate factors in the association between urine metals and ePWV

To explore potential underlying mechanisms between metal exposure and ePWV, a parallel mediation analysis was conducted to evaluate the mediating effect of serum lipid levels and white blood cell counts on the aforementioned association. Parallel mediation analysis demonstrated that HDL and TC exerted a weak but significant mediation effect on the association between mixed metals WQS indices and ePWV, and the proportion of mediation was 2.14 and 3.70% (Table 2). The mediated proportion of HDL on the associations between Co, Cs, Pb, U, As and ePWV was 1.50, 5.73, 2.29, 5.29 and 18.82% (*p* < 0.05). TC similarly mediated the association between Ba, Cd, Co, Cs, Pb, U and ePWV, and the proportion of mediation was 11.88, 6.44, −6.04%, 5.55, 4.91 and 6.40% (Supplementary Table S4).

**Table 2:** Mediation analysis on the association of multiple metals WQS index with ePWV.

Mediators	Indirect effects	Direct effects	Total effects	Mediated proportion (%)	*p*-value
	*β* (95% CI)	*β* (95% CI)	*β* (95% CI)		
HDL	0.0006 (0.0002, 0.0009)	0.027 (0.023, 0.031)	0.028 (0.023, 0.032)	2.14%	<0.001
TC	0.001 (0.0007, 0.002)	0.026 (0.022, 0.030)	0.027 (0.024, 0.031)	3.70%	<0.001
WBC	0.001 (−0.002, 0.003)	0.026 (0.022, 0.031)	0.026 (0.022, 0.031)	-	0.892
LYM	−0.001 (−0.001, 0.0001)	0.026 (0.022, 0.031)	0.026 (0.022, 0.031)	-	0.732
MON	0.001 (−0.001, 0.001)	0.026 (0.022, 0.031)	0.026 (0.022, 0.031)	-	0.866
NEU	−0.001 (−0.001, 0.001)	0.026 (0.022, 0.031)	0.026 (0.022, 0.031)	-	0.726
EOS	−0.001 (−0.002, 0.001)	0.027 (0.022, 0.031)	0.026 (0.022, 0.031)	-	0.238
BAS	−0.001 (−0.001, 0.001)	0.026 (0.022, 0.031)	0.026 (0.022, 0.031)	-	0.900

Model was adjusted for age, gender, race, education levels, smoking status, past-year alcohol drinking, intake of total energy, physical activity, BMI (body mass index), ratio of family income to poverty, diabetes, hypertension, CVD (cardiovascular diseases), NHANES survey circle, level of high-density lipoprotein and total cholesterol (apart from the analyses of HDL and TC).

ePWV, Estimated Pulse Wave Velocity. WBC, White blood cell count. LYM, Lymphocyte number. MON, Monocyte number. NEU, Segmented neutrophils number. EOS, Eosinophils number. BAS, Basophils number. HDL, high-density lipoprotein. TC, total cholesterol.

## 4 Discussion

In this study, we observed an association between metal exposure and ePWV based on the NHANES cohort. In the multiple linear regression analysis, higher levels of urine U, Pb, Cd and Co were related to increased ePWV. Mixture analysis models revealed a significant correlation between metal mixture and elevated ePWV, with Pb and Cd as the primary contributors. Furthermore, mediation analysis indicated that HDL and TC accounted for a mediation effect of 2.14 and 3.70% on the association of multiple metals WQS index with ePWV.

Metals are widely used in industrial production and are therefore released into the environment. The general population is mainly exposed to metals through air, food, and drinking water, while occupational exposure exists in mining, smelting, and chemical manufacturing industries (13). Considering the complexity of real-world scenarios, individuals are always exposed to multiple metals. We further used the WQS and Qgcomp models to evaluate the collective effects of multiple metals. Based on the aforementioned models, we found that mixed metal exposure increases the risk of arterial stiffness. This finding is supported by a multicenter study involving multiple ethnic groups, where higher levels of urine metals were linked to the progression of coronary artery stiffness (26). Furthermore, we identified that urine Cd and Pb contributed most to the elevated ePWV through a mixture analysis model. These findings provide a basis for reducing related metals exposure. Additionally, we observed that the WQS regression revealed a significant association between U and increased ePWV, whereas the conclusion of Qgcomp was different. This phenomenon may be attributed to the nature that WQS regression is restricted to gaging risk factors in the same direction (27).

Our mediation analysis indicate that HDL/TC play a promotional role in the association between metal exposure and elevated ePWV. Substantial evidence implicates Pb and Cd could elevate TC level, as well as reduced HDL level (28, 29). HDL and TC were also associated with baPWV/cfPWV (30, 31). Preclinical studies in animal models suggests that metal exposure can disrupt lipid metabolism (32). These evidences indicate that Pb and Cd can disrupt the biological activity of enzymes involved in lipid metabolism, leading to lipid metabolism disorders and promoting arterial stiffness.

However, it should be emphasized that although the mediating effect of HDL and TC was significant, its proportion of total effect was faint in our model. These findings suggest that the mechanism underlying the metal-induced arterial stiffness remains to be elucidated. However, the findings from animal studies may offer some clues. Pb and Cd exposure can reduce NO production by inhibiting eNOS (33, 34), augment the secretion levels of endothelial cell adhesion molecule (35, 36), eventually resulting in endothelial dysfunction and vascular injury. Furthermore, Pb and Cd may also compete with calcium in calcium-dependent processes and interact with calmodulin, thereby disrupting calcium homeostasis and inducing smooth muscle contraction and relaxation dysfunction (37).

This study highlights several notable advantages. First, we conducted a comprehensive population-based analysis to ascertain the role of metal exposure in the progression of arterial stiffness, providing important corroborations with regard to the cardiovascular consequences induced by metal exposure. Second, we employed two mixture analysis models to simulate real-world scenarios and discriminate high-risk metals. Third, we explored potential mechanisms through mediation analysis, suggesting that metal exposure may exacerbate arterial stiffness through perturbations in lipid homeostasis. The findings presented herein provide indispensable epidemiological evidence for identifying the vascular impairment caused by metal exposure. It also indicates that policymakers should take measures to mitigate and control sources of exposure, which is crucial to reducing metal contamination in air, water, and food, thereby ensuring public health.

A few limitations should be acknowledged. First, the cross-sectional nature of our study design limits the capacity to establish a causal relationship between metal exposure and arterial stiffness. Second, given the disparity in half-life and distribution between metals, urine metal levels may not accurately represent actual exposure *in vivo*. Third, our analyses did not consider sampling weights, which may have impacted the conclusions. In conclusion, it is imperative that the findings pertaining to the association between metal exposure and arterial stiffness are corroborated in forthcoming experimental studies and prospective studies with augmented sample sizes.

## 5 Conclusion

Our research provides the following findings. First, mixed and single exposure model emphasized that metal exposure increases the risk of high ePWV level, especially Pb and Cd. Furthermore, HDL and TC may act as mediating factors for the associations of metal exposure with elevated ePWV, though this mediating effect is negligible.

## Data Availability

Publicly available datasets were analyzed in this study. This data can be found at: https://www.cdc.gov/nchs/nhanes/.

## Footnotes

^1^ https://www.cdc.gov/nchs/nhanes/

## Glossary

ePWV: estimate pulse wave velocity
cfPWV: carotid-femoral pulse wave velocity
NHANES: National Health and Nutrition Examination Survey
WQS: Weighted Quantile Sum regression
Qgcomp: Quantile g-computation
RCS: restricted cubic spline regression
PIR: poverty income ratio
Sb: antimony
As: arsenic
Ba: barium
Cd: cadmium
Cs: cesium
Co: cobalt
Pb: lead
Mo: molybdenum
Hg: mercury
TI: thallium
W: tungsten
U: uranium
HDL: high-density lipoprotein
TC: total cholesterol
MBP: mean blood pressure
SBP: systolic blood pressure
DBP: diastolic blood pressure

## References

[1] Mitchell GF Hwang S-J Vasan RS Larson MG Pencina MJ Hamburg NM. Arterial stiffness and cardiovascular events: the Framingham heart study. Circulation. (2010) 121:505–11. doi: 10.1161/CIRCULATIONAHA.109.886655, PMID: 20083680 PMC2836717

[2] Hill MA Yang Y Zhang L Sun Z Jia G Parrish AR. Insulin resistance, cardiovascular stiffening and cardiovascular disease. Metabolism. (2021) 119:154766. doi: 10.1016/j.metabol.2021.154766, PMID: 33766485

[3] Tan L Liu Y Liu J Zhang G Liu Z Shi R. Association between insulin resistance and uncontrolled hypertension and arterial stiffness among US adults: a population-based study. Cardiovasc Diabetol. (2023) 22:311. doi: 10.1186/s12933-023-02038-5, PMID: 37946205 PMC10637002

[4] Fiori G Fuiano F Scorza A Conforto S Sciuto SA. Non-invasive methods for PWV measurement in blood vessel stiffness assessment. IEEE Rev Biomed Eng. (2021) 15:169–83. doi: 10.1109/RBME.2021.3092208 34166202

[5] Germano G Hoes A Karadeniz S Mezzani A Prescott E Ryden L. European guidelines on cardiovascular disease prevention in clinical practice (version 2012). Eur Heart J. (2012) 33:1635–701. doi: 10.1093/eurheartj/ehs092 22555213

[6] Terentes-Printzios D Vlachopoulos C. Arterial stiffness for cardiovascular risk stratification in clinical practice In: Julio AC, editor. Textbook of arterial stiffness and pulsatile hemodynamics in health and disease. Amsterdam, Netherlands: Elsevier (2022). 503–25.

[7] Rey-García J Townsend RR. Large artery stiffness: a companion to the 2015 AHA science statement on arterial stiffness. Pulse. (2021) 9:1–10. doi: 10.1159/000518613, PMID: 34722350 PMC8527919

[8] Greve SV Blicher MK Kruger R Sehestedt T Gram-Kampmann E Rasmussen S. Estimated carotid–femoral pulse wave velocity has similar predictive value as measured carotid–femoral pulse wave velocity. J Hypertens. (2016) 34:1279–89. doi: 10.1097/HJH.0000000000000935 27088638

[9] Vlachopoulos C Terentes-Printzios D Laurent S Nilsson PM Protogerou AD Aznaouridis K. Association of estimated pulse wave velocity with survival: a secondary analysis of SPRINT. JAMA Netw Open. (2019) 2:e1912831–1. doi: 10.1001/jamanetworkopen.2019.12831, PMID: 31596491 PMC6802234

[10] Solini A Orsi E Vitale M Garofolo M Resi V Bonora E. Independent association of estimated pulse-wave velocity with all-cause mortality in individuals with type 2 diabetes. QJM Int J Med. (2024) 117:hcae012. doi: 10.1093/qjmed/hcae012, PMID: 38200621

[11] Wei J Li H Liu J. Heavy metal pollution in the soil around municipal solid waste incinerators and its health risks in China. Environ Res. (2022) 203:111871. doi: 10.1016/j.envres.2021.111871, PMID: 34390720

[12] Qin G Niu Z Yu J Li Z Ma J Xiang P. Soil heavy metal pollution and food safety in China: effects, sources and removing technology. Chemosphere. (2021) 267:129205. doi: 10.1016/j.chemosphere.2020.129205, PMID: 33338709

[13] Tchounwou PB Yedjou CG Patlolla AK Sutton DJ. Heavy metal toxicity and the environment. Exp Suppl. (2012) 101:133–64. doi: 10.1007/978-3-7643-8340-4_6 22945569 PMC4144270

[14] Tian W Zhang M Zong D Li W Li X Wang Z. Are high-risk heavy metal (loid) s contaminated vegetables detrimental to human health? A study of incorporating bioaccessibility and toxicity into accurate health risk assessment. Sci Total Environ. (2023) 897:165514. doi: 10.1016/j.scitotenv.2023.165514, PMID: 37451464

[15] Lamas GA Bhatnagar A Jones MR Mann KK Nasir K Tellez-Plaza M. Contaminant metals as cardiovascular risk factors: a scientific statement from the American Heart Association. J Am Heart Assoc. (2023) 12:e029852. doi: 10.1161/JAHA.123.029852, PMID: 37306302 PMC10356104

[16] Xie J Zhou F Ouyang L Li Q Rao S Su R. Insight into the effect of a heavy metal mixture on neurological damage in rats through combined serum metabolomic and brain proteomic analyses. Sci Total Environ. (2023) 895:165009. doi: 10.1016/j.scitotenv.2023.165009, PMID: 37353033

[17] Li K Wu J Mei Y Zhao J Zhou Q Li Y. Metallomics analysis of metal exposure and cognitive function in older adults: a combined epidemiological and bioinformatics study. Chemosphere. (2023) 341:140049. doi: 10.1016/j.chemosphere.2023.140049, PMID: 37660799

[18] Tang M Zhao Y Zhai Y Zhang Y Liu Y Liu T. Mercury chloride activates the IFNγ-IRF1 signaling in myeloid progenitors and promotes monopoiesis in mice. Environ Pollut. (2023) 337:122583. doi: 10.1016/j.envpol.2023.122583, PMID: 37741541

[19] Lu Y Wu J Gu W Huang Z Shu Z Huang M. Single-cell transcriptomics uncovers phenotypic alterations in the monocytes in a Chinese population with chronic cadmium exposure. Ecotoxicol Environ Saf. (2021) 211:111881. doi: 10.1016/j.ecoenv.2020.111881, PMID: 33444878

[20] Zhao Y He J Zhu T Zhang Y Zhai Y Xue P. Cadmium exposure reprograms energy metabolism of hematopoietic stem cells to promote myelopoiesis at the expense of lymphopoiesis in mice. Ecotoxicol Environ Saf. (2022) 231:113208. doi: 10.1016/j.ecoenv.2022.113208 35051759

[21] Wan Z Wu M Liu Q Fan G Fang Q Qin X. Association of metal exposure with arterial stiffness in Chinese adults. Ecotoxicol Environ Saf. (2023) 257:114921. doi: 10.1016/j.ecoenv.2023.114921, PMID: 37080131

[22] Li P Ma J Jiang Y Yang X Luo Y Tao L. Association between mixed heavy metal exposure and arterial stiffness, with alkaline phosphatase identified as a mediator. Biol Trace Elem Res. (2025) 203:3457–69. doi: 10.1007/s12011-024-04359-2, PMID: 39218814

[23] Wang X Gao D Zhang G Zhang X Li Q Gao Q. Exposure to multiple metals in early pregnancy and gestational diabetes mellitus: a prospective cohort study. Environ Int. (2020) 135:105370. doi: 10.1016/j.envint.2019.105370, PMID: 31864020

[24] Liu Q Jin J Xu C Li W Liang J Xu J. HDL cholesterol: a potential mediator of the association between serum levels of a mixture of metals and the risk of aortic dissection in a Chinese population. Environ Pollut. (2021) 290:117942. doi: 10.1016/j.envpol.2021.117942, PMID: 34454198

[25] Huang X Wu Y Lu Y. Single and mixed effects of seven heavy metals on stroke risk: 11,803 adults from National Health and nutrition examination survey (NHANES). Front Nutr. (2025) 12:1524099. doi: 10.3389/fnut.2025.1524099, PMID: 40144574 PMC11937853

[26] McGraw KE Schilling K Glabonjat RA Galvez-Fernandez M Domingo-Relloso A Martinez-Morata I. Urinary metal levels and coronary artery calcification: longitudinal evidence in the multi-ethnic study of atherosclerosis. J Am Coll Cardiol. (2024) 84:1545–57. doi: 10.1016/j.jacc.2024.07.020, PMID: 39297845 PMC11804863

[27] Guo X Wu B Hu W Wang X Su W Meng J. Associations of perchlorate, nitrate, and thiocyanate with metabolic syndrome and its components among US adults: a cross-sectional study from NHANES. Sci Total Environ. (2023) 879:163083. doi: 10.1016/j.scitotenv.2023.163083, PMID: 36972877

[28] Zhao M Yin G Xu J Ge X Li A Mei Y. Independent, combine and interactive effects of heavy metal exposure on dyslipidemia biomarkers: a cross-sectional study in northeastern China. Ecotoxicol Environ Saf. (2023) 250:114494. doi: 10.1016/j.ecoenv.2022.114494, PMID: 36608569

[29] Kim DW Ock J Moon KW Park CH. Association between heavy metal exposure and dyslipidemia among Korean adults: from the Korean National Environmental Health Survey, 2015-2017. Int J Environ Res Public Health. (2022) 19:181. doi: 10.3390/ijerph19063181, PMID: 35328872 PMC8951064

[30] Chen C Dai JL. Triglyceride to high-density lipoprotein cholesterol (HDL-C) ratio and arterial stiffness in Japanese population: a secondary analysis based on a cross-sectional study. Lipids Health Dis. (2018) 17:130. doi: 10.1186/s12944-018-0776-7, PMID: 29843793 PMC5975424

[31] Agbaje AO Lloyd-Jones DM Magnussen CG Tuomainen TP. Cumulative dyslipidemia with arterial stiffness and carotid IMT progression in asymptomatic adolescents: a simulated intervention longitudinal study using temporal inverse allocation model. Atherosclerosis. (2023) 364:39–48. doi: 10.1016/j.atherosclerosis.2022.11.011, PMID: 36462968

[32] Hong H Xu Y Xu J Zhang J Xi Y Pi H. Cadmium exposure impairs pancreatic β-cell function and exaggerates diabetes by disrupting lipid metabolism. Environ Int. (2021) 149:106406. doi: 10.1016/j.envint.2021.106406, PMID: 33508533

[33] Gonick HC Ding Y Bondy SC Ni Z Vaziri ND. Lead-induced hypertension: interplay of nitric oxide and reactive oxygen species. Hypertension. (1997) 30:1487–92. doi: 10.1161/01.hyp.30.6.1487, PMID: 9403571

[34] Majumder S Muley A Kolluru GK Saurabh S Tamilarasan KP Chandrasekhar S. Cadmium reduces nitric oxide production by impairing phosphorylation of endothelial nitric oxide synthase. Biochem Cell Biol. (2008) 86:1–10. doi: 10.1139/o07-146, PMID: 18364740

[35] Messner B Knoflach M Seubert A Ritsch A Pfaller K Henderson B. Cadmium is a novel and independent risk factor for early atherosclerosis mechanisms and in vivo relevance. Arterioscler Thromb Vasc Biol. (2009) 29:1392–8. doi: 10.1161/ATVBAHA.109.190082, PMID: 19556524

[36] Camaj PR Graziano JH Preteni E Popovac D LoIacono N Balac O. Long-term effects of environmental Lead exposure on blood pressure and plasma soluble cell adhesion molecules in young adults: a follow-up study of a prospective cohort in Kosovo. J Environ Public Health. (2018) 2018:1–10. doi: 10.1155/2018/3180487, PMID: 29535789 PMC5817317

[37] Hechtenberg S Beyersmann D. Inhibition of sarcoplasmic reticulum ca(2+)-ATPase activity by cadmium, lead and mercury. Enzyme. (1991) 45:109–15. doi: 10.1159/000468875, PMID: 1840035

