# Supplementary material for: Multiple metals exposure and estimated pulse wave velocity: a cross-sectional analysis of the US adults
Source: Front Public Health. 2025 Aug 26;13:1606518. doi: 10.3389/fpubh.2025.1606518 (PMC12419226; doi:10.3389/fpubh.2025.1606518)
Supplement: Supplementary file 1 [file Data_Sheet_1.docx]

**Multiple metal exposures and Estimated Pulse Wave Velocity: A cross-sectional analysis of the National Health and Nutrition Examination Survey 2003-2016**

**Table of Contents**

**Supplemental Table 1.** Basic information of environmental metal contaminants.

**Supplemental Table 2.** The distributions of environmental metal exposure among the study participants.

**Supplemental Table 3.** The association between environmental metal exposures with ePWV.

**Supplemental Table 4.** Mediation analysis on the association of single metal exposure with ePWV.

**Supplemental Figure 1.** The Spearman's correlation analysis for the mutual correlations among the environmental metal exposures.

**Table S1.** Basic information of environmental metal contaminants.

| Metals | Abbreviation | LLOD (μg/L) | Detection rate |
| --- | --- | --- | --- |
| Barium | Ba | 0.060 | 99.5% |
| Cadmium | Cd | 0.036 | 79.8% |
| Cobalt | Co | 0.023 | 99.7% |
| Cesium | Cs | 0.086 | 99.8% |
| Molybdenum | Mo | 0.800 | 99.8% |
| Lead | Pb | 0.030 | 97.5% |
| Antimony | Sb | 0.022 | 69.4% |
| Thallium | Tl | 0.018 | 99.3% |
| Tungsten | W | 0.018 | 85.1% |
| Uranium | U | 0.002 | 78.2% |
| Arsenic | As | 0.26 | 99.5% |
| Mercury | Hg | 0.13 | 80.9% |

LLOD, lower limit of detection.

**Table S2.** The distributions of environmental metal exposure among the study participants.

| Variable  (μg/mg Cr) | P10 | P25 | P50 | P75 | P90 | GM (GSD) |
| --- | --- | --- | --- | --- | --- | --- |
| Ba | 0.037 | 0.067 | 0.122 | 0.224 | 0.395 | 0.137 (2.339) |
| Cd | 0.008 | 0.014 | 0.025 | 0.045 | 0.076 | 0.038 (1.822) |
| Co | 0.016 | 0.023 | 0.033 | 0.051 | 0.083 | 0.047 (1.733) |
| Cs | 0.237 | 0.315 | 0.426 | 0.595 | 0.815 | 0.444 (1.632) |
| Mo | 1.784 | 2.634 | 3.875 | 5.796 | 8.530 | 3.935 (1.915) |
| Pb | 0.019 | 0.029 | 0.048 | 0.080 | 0.126 | 0.062 (1.858) |
| Sb | 0.003 | 0.004 | 0.006 | 0.009 | 0.014 | 0.017 (1.363) |
| Tl | 0.008 | 0.011 | 0.015 | 0.021 | 0.029 | 0.026 (1.391) |
| W | 0.002 | 0.004 | 0.007 | 0.011 | 0.020 | 0.018 (1.521) |
| U | 0.0002 | 0.0004 | 0.0006 | 0.0009 | 0.002 | 0.011 (1.150) |
| As | 0.287 | 0.432 | 0.744 | 1.511 | 3.361 | 0.886 (2.691) |
| Hg | 0.010 | 0.018 | 0.036 | 0.072 | 0.135 | 0.051 (2.159) |

Cr, urine creatinine. P, percentile. GM, geometric mean. GSD, geometric standard deviation. Ba, Barium. Cd, Cadmium. Co, Cobalt. Cs, Cesium. Mo, Molybdenum. Pb, Lead. Sb, Antimony. Tl, Thallium. W, Tungsten. U, Uranium. As, Arsenic. Hg, Mercury.

**Table S3.** The association between environmental metal exposures with ePWV.

|  | ePWV, β (95%CI) | | | | | | | | | | | |
| --- | --- | --- | --- | --- | --- | --- | --- | --- | --- | --- | --- | --- |
|  | Ba | Cd | Co | Cs | Mo | Pb | Sb | Tl | W | U | As | Hg |
| Total population | 0.002  (-0.002, 0.005) | **0.012**  **(0.006, 0.017)** | **0.012**  **(0.006, 0.017)** | **0.008**  **(0.001, 0.015)** | 0.003  (-0.002 0.007) | **0.024**  **(0.019, 0.029)** | **-0.009**  **(-0.018, -0.001)** | **-0.015**  **(-0.024, -0.005)** | **-0.008**  **(-0.015, -0.002)** | **0.031**  **(0.012, 0.051)** | 0.001  (-0.002, 0.003) | **-0.005**  **(-0.009, -0.002)** |
| Age |  |  |  |  |  |  |  |  |  |  |  |  |
| <40 | 0.004  (-0.001, 0.008) | **0.014**  **(0.006, 0.021)** | 0.006  (-0.002, 0.014) | 0.008  (-0.001, 0.016) | -0.008  (-0.013, 0.002) | -0.001  (-0.008, 0.005) | 0.002  (-0.008, 0.012) | -0.005  (-0.017, 0.007) | 0.006  (-0.001, 0.013) | 0.012  (-0.010, 0.035) | -0.001  (-0.005, 0.002) | -0.001  (-0.006, 0.003) |
| 40-60 | **0.007**  **(0.001, 0.014** | **0.021**  **(0.011, 0.031** | -0.004  (-0.014 0.006) | **0.014**  **(0.002, 0.027)** | -0.003  (-0.011 0.005) | **0.037**  **(0.028, 0.047)** | -0.005  (-0.020, 0.009) | **-0.017**  **(-0.033, -0.001)** | -0.010  (-0.022, 0.002) | **0.041**  **(0.004, 0.078)** | 0.001  (-0.005, 0.005) | -0.004  (-0.010, 0.003) |
| >60 | -0.003  (-0.009, 0.004) | -0.005  (-0.015, 0.005) | **0.031**  **(0.022, 0.040)** | 0.005  (-0.008, 0.018) | **0.011**  **(0.003, 0.018)** | **0.024**  **(0.014, 0.034)** | **-0.022**  **(-0.039, -0.005)** | -0.015  (-0.032, 0.002) | **-0.025**  **(-0.038, -0.013)** | **0.035**  **(0.001, 0.069)** | 0.003  (-0.002, 0.008) | **-0.009**  **(-0.017, -0.002)** |
| Gender |  |  |  |  |  |  |  |  |  |  |  |  |
| Male | -0.002  (-0.007, 0.003) | **0.010**  **(0.002, 0.018)** | **0.019**  **(0.011, 0.028)** | 0.001  (-0.009, 0.010) | 0.003  (-0.003, 0.009) | **0.023**  **(0.016, 0.030)** | **-0.017**  **(-0.029, -0.006)** | -0.012  (-0.026, 0.002) | -0.006  (-0.016, 0.003) | **0.039**  **(0.011, 0.069)** | 0.002  (-0.002, 0.005) | -0.005  (-0.011, 0.001) |
| Female | 0.004  (-0.001, 0.009) | **0.014**  **(0.006, 0.021)** | **0.011**  **(0.003, 0.018)** | **0.015**  **(0.005, 0.025)** | 0.002  (-0.005, 0.008) | **0.024**  **(0.016, 0.032)** | -0.003  (-0.016, 0.011) | -0.019  (-0.032, -0.005) | **-0.010**  **(-0.020, -0.001)** | 0.023  (-0.003, 0.049) | -0.001  (-0.005, 0.004) | -0.005  (-0.010, 0.001) |
| Race |  |  |  |  |  |  |  |  |  |  |  |  |
| White | 0.000  (-0.005, 0.005) | 0.006  (-0.002, 0.015) | **0.017**  **(0.009, 0.024)** | 0.000  (-0.010, 0.010) | 0.003  (-0.004, 0.009) | **0.037**  **(0.029, 0.045)** | **-0.015**  **(-0.027, -0.002)** | -0.008  (-0.022, 0.007) | **-0.014**  **(-0.024, -0.004)** | **0.036**  **(0.010, 0.062)** | 0.003  (-0.002, 0.007) | -0.001  (-0.006, 0.005) |
| Non-White | 0.004  (-0.001, 0.008) | **0.021**  **(0.013, 0.028)** | -0.001  (-0.009, 0.007) | **0.010**  **(0.001, 0.019)** | -0.001  (-0.006, 0.006) | **0.012**  **(0.005, 0.019)** | -0.007  (-0.019, 0.005) | **-0.013**  **(-0.026, -0.001)** | -0.002  (-0.011, 0.007) | 0.020  (-0.008, 0.048) | -0.001  (-0.004, 0.003) | **-0.012**  **(-0.017, -0.006)** |

The multiple linear regression models were adjusted for age, gender, race, education levels, smoking status, past-year alcohol drinking, intake of total energy, physical activity, BMI(body mass index), ratio of family income to poverty, diabetes, hypertension, CVD(cardiovascular diseases), NHANES survey circle, level of high-density lipoprotein and total cholesterol.

ePWV, Estimated Pulse Wave Velocity. Ba, Barium. Cd, Cadmium. Co, Cobalt. Cs, Cesium. Mo, Molybdenum. Pb, Lead. Sb, Antimony. Tl, Thallium. W, Tungsten. U, Uranium. As, Arsenic. Hg, Mercury.

**Table S4.** Mediation analysis on the association of single metal exposure with ePWV.

| Mediators | Exposure | Indirect effects | Direct effects | Total effects | Mediated proportion | *P* value |  |
| --- | --- | --- | --- | --- | --- | --- | --- |
|  |  | β (95%CI) | β (95%CI) | β (95%CI) | (%) |  |  |
| HDL |  |  |  |  |  |  |  |
|  | Ba | 0.001(-0.001, 0.001) | 0.009(0.006, 0.012) | 0.009(0.006, 0.012) | . | 0.230 |  |
|  | Cd | 0.001(-0.001, 0.001) | 0.018(0.013, 0.024) | 0.018(0.013, 0.024) | . | 0.642 |  |
|  | Co | 0.0003(0.00002, 0.0006) | 0.017(0.012, 0.022) | 0.017(0.012, 0.022) | 1.50% | 0.024 |  |
|  | Cs | 0.0007(0.0004, 0.0013) | 0.013(0.007, 0.019) | 0.014(0.008, 0.019) | 5.73% | <0.001 |  |
|  | Mo | 0.001(-0.001, 0.001) | 0.004(-0.001, 0.008) | 0.003(-0.001, 0.007) | . | 0.104 |  |
|  | Pb | 0.0007(0.0003, 0.0011) | 0.029(0.024, 0.034) | 0.030(0.025, 0.034) | 2.29% | <0.001 |  |
|  | Sb | 0.001(0.0005, 0.002) | 0.003(-0.006, 0.011) | 0.004(-0.005, 0.012) | . | 0.390 |  |
|  | Tl | 0.001(0.001, 0.002) | 0.002(-0.005, 0.010) | 0.004(-0.004, 0.012) | . | 0.356 |  |
|  | W | 0.001(-0.001, 0.001) | -0.003(-0.009, 0.003) | -0.003(-0.009, 0.003) | . | 0.890 |  |
|  | U | 0.002(0.001, 0.004) | 0.040(0.021, 0.060) | 0.043(0.023, 0.062) | 5.29% | <0.001 |  |
|  | As | 0.001(0.0003, 0.001) | 0.002(-0.001, 0.005) | 0.003(0.0002, 0.006) | 18.82% | 0.038 |  |
|  | Hg | 0.001(0.0004, 0.001) | -0.004(-0.007, -0.00009) | -0.003(-0.007, 0.001) | . | 0.108 |  |
| TC |  |  |  |  |  |  |  |
|  | Ba | 0.0010(0.0006, 0.0014) | 0.008(0.005, 0.011) | 0.009(0.006, 0.012) | 11.28% | <0.001 |  |
|  | Cd | 0.0012(0.0005, 0.0018) | 0.017(0.011, 0.022) | 0.018(0.013, 0.024) | 6.44% | <0.001 |  |
|  | Co | -0.001(-0.002, -0.0005) | 0.018(0.013, 0.023) | 0.017(0.012, 0.022) | -6.04% | <0.001 |  |
|  | Cs | 0.0008(0.00008, 0.0014) | 0.013(0.007, 0.019) | 0.014(0.008, 0.020) | 5.55% | 0.028 |  |
|  | Mo | -0.001(-0.002, -0.001) | 0.005(0.001, 0.009) | 0.003(-0.001, 0.008) | . | 0.098 |  |
|  | Pb | 0.0015(0.0009, 0.0021) | 0.028(0.023, 0.033) | 0.030(0.025, 0.034) | 4.91% | <0.001 |  |
|  | Sb | 0.0004(-0.001, 0.001) | 0.004(-0.004, 0.012) | 0.004(-0.004, 0.013) | . | 0.650 |  |
|  | Tl | 0.002(0.001, 0.003) | 0.002(-0.005, 0.010) | 0.004(-0.003, 0.012) | . | 0.350 |  |
|  | W | -0.0008(-0.0015, -0.00005) | -0.002(-0.008, 0.004) | -0.003(-0.009, 0.003) | . | 0.392 |  |
|  | U | 0.003(0.00004, 0.005) | 0.039(0.019, 0.058) | 0.042(0.022, 0.06) | 6.40% | 0.018 |  |
|  | As | 0.0004(0.00004, 0.001) | 0.002(-0.001, 0.005) | 0.003(0.00008, 0.006) | . | 0.078 |  |
|  | Hg | 0.001(0.0002, 0.001) | -0.004(-0.007, -0.0001) | -0.003(-0.006, 0.001) | . | 0.110 |  |

Model was adjusted for age, gender, race, education levels, smoking status, past-year alcohol drinking, intake of total energy, physical activity, BMI(body mass index), ratio of family income to poverty, diabetes, hypertension, CVD(cardiovascular diseases), NHANES survey circle.

ePWV, Estimated Pulse Wave Velocity. HDL, high-density lipoprotein. TC, total cholesterol. Ba, Barium. Cd, Cadmium. Co, Cobalt. Cs, Cesium. Mo, Molybdenum. Pb, Lead. Sb, Antimony. Tl, Thallium. W, Tungsten. U, Uranium. As, Arsenic. Hg, Mercury.


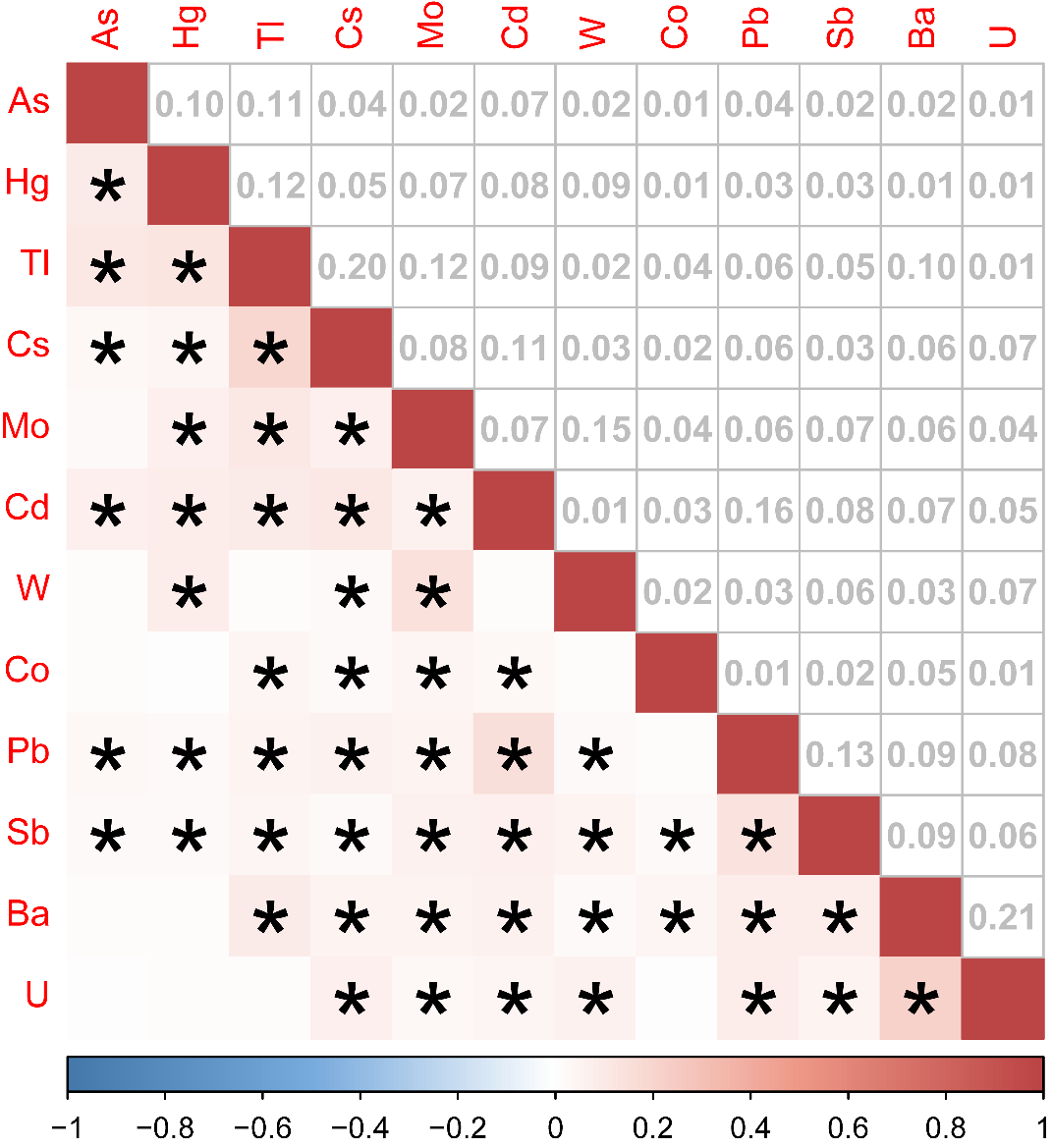


**Figure S1.** The Spearman's correlation analysis for the mutual correlations among the environmental metal exposures.
